# Race science without racists: how bigoted paradigms persist in allergy research

**DOI:** 10.3389/fpubh.2024.1351732

**Published:** 2024-07-10

**Authors:** Ian A. Myles

**Affiliations:** Laboratory of Clinical Immunology and Microbiology, Epithelial Therapeutics Unit, National Institute of Allergy and Infectious Disease, National Institutes of Health, Bethesda, MD, United States

**Keywords:** racism, race science, eczema, atopic dermatitis, allergy

## Abstract

In the wake of the murder of George Floyd and the massacre in Buffalo, the editorial boards of the prominent scientific publication companies formally apologized for their journals’ historical role in advancing race science and promised to improve their standards. However, flowery commentaries cannot undo the consistent pattern of endorsing biologic differences between ethnic groups, even when discussing diseases or traits that are not considered politically charged. In this report, an exemplar is made of a recent publication claiming to identify phenotypes of atopic dermatitis that are distinct between European Americans, Asians, and African Americans. The insufficiency of the evidence and logic underlying these claims are discussed. Although devoid of malice, numerous publications continue to demonstrate how claims of biological differences between races is mainstreamed in modern scientific publications. Overall, the goal of this work is to challenge the scientific community, particularly the publication companies, to evaluate how assumptions of innate biologic disadvantage have clouded assessments of racial disparities in disease beyond the topics that are more stereotypical of race science.

## 1 Introduction

In the wake of the murder of George Floyd and the massacre in Buffalo, *Nature* (Springer-Nature) editors formally apologized for their journals’ historical role in advancing race science (1) and promised to improve their standards (2). Other prominent journals such as *Science* (AAAS) and *Cell* (Elsevier) also appeared to grapple with their pasts (3–5). However well intentioned, such commentaries failed to address the continued pattern of endorsing biologic differences between ethnic groups, even when discussing diseases or traits that are not considered politically charged. A case study in this practice is presented by the recent work from Facheris et al. entitled “The translational revolution in atopic dermatitis: the paradigm shift from pathogenesis to treatment” (6). This manuscript appeared in *Cellular & Molecular Immunology* (a journal within the Springer-Nature family). Sadly, the work presented is more representative of an entrenched status quo than Thomas Kuhn’s intended definition of a shifting paradigm (7).

## 2 How race science paradigms persist

### 2.1 Conflating racial claims with ancestral categories

While the paper by Facheris et al. nicely outlines the targeted treatments in development for atopic dermatitis (AD), immune modulation for immune mediated diseases is not a paradigm shift. Yet while focusing on presenting AD from the perspective of cytokine imbalance, the authors also feed into the pattern of unintentional endorsement of race science that *Nature* assured its readers it would attempt to avoid (2). The authors propose generalized differences between ancestries (6, 8) exist in the molecular pathology underlying AD symptoms using primary citations which exclusively rely on racial categorization. For example, the authors claim to have “confirmed” a patient’s ancestry in a clinic visit (8) when only self-reported race or ethnicity could be assessed in such a manner. The racialized AD claims in Facheris, *et al* appear to have been initially presented in Czarnowski et al. 4 years prior (9). While the discussion herein focuses on the Facheris et al. and Czarnowski et al. publications in lieu of other examples (10–14), the aim is not to deride specific manuscripts but to dissect them for a teachable moment of how entrenched claims of racialized biologic determinism are unintentionally perpetuated in the scientific literature due to an uncritical assessment of the underlying evidence.

### 2.2 Overextrapolation from small, unrelated studies

Using the *de facto* racial and ethnic categorizations, both Facheris and Czarnowski claim to have found molecular and biochemical classifications of AD unique to European Americans, Asians, and African Americans. Based on the citations provided by the authors, the claims of distinguishing immunologic markers between European and Asians AD were derived by contrasting two different studies, by two different groups, using different assay equipment, in two different countries, at two different points in time. Claims of Asian-specific AD was based only comparing the cytokine profiles from a Japanese population with AD (*n* = 42) (15) vs. a separate cohort of 51 European American (16, 17). The imperfect overlap was summarized as “European American AD cohorts feature relatively high activity of the Th2 and Th22 axes … compared to Asian and African American Cohorts” (6, 8). The only other set of studies presented as support for the existence of “the Asian AD cohort” included 21 patients “of Han Chinese descent” juxtaposed against an unrelated retrospective analysis of 107 European Americans (many of whom were both over 65 years of age and hospitalized) (18, 19).

Only one study of 15 Black and 15 White Manhattanites was used to support claims that “African American AD cohorts are characterized by an absence of Th17…” (8). Although a similar study in 18 Black Marylanders found ample Th17 signal (14), both studies failed to document a single social determinant or any of the numerous AD risk factors known to be unevenly distributed between racial groups (20). This practice is inconsistent with *Nature*’s updated policies on the use of racial categorization devoid of statistical adjustments for environmental exposures and social determinants of health (21).

### 2.3 Failure to assess for environmental factors or social determinants of health

A non-exhaustive list of exposures linked to AD would include at least the nation of birth, urbanicity of residence, distance from a major roadway, the number and type of animals and siblings living in the home, as well as exposure to: traffic related air pollution, diisocyanates, particulate matter under 2.5 microns (PM_2.5_), nitric oxide, hard water, phthalates, early life antibiotics, synthetic fabrics, cigarette smoke, as well as foods low in fiber or high in refined ingredients such as saturated fat, refined sugar, surfactants, oxidizers, and emulsifiers (22–38). Most of these exposures are not evenly distributed across racial groups (20). Beyond overt environmental injustices, racial differences in skin health could stem from differences in skincare product choice (39) or access to AD medications (40). Each of these factors should be assessed prior to making any racialized conclusions in data.

### 2.4 Is innate biology a sound hypothesis for explaining racial disparities in AD?

The perceived racial difference in AD manifestation seems limited to the phenotypic presentation of active lesions. While most image atlases of AD are skewed toward visuals of the manifestations in skin of Caucasians, online tools have been developed (41) to provide clinicians with examples of the variable presentation of AD in different skin types (accessible through the National Eczema Association).^1^ However, if skin pigmentation directly impacts AD risk (as opposed to being a marker of environmental injustice) one must explain the disparities *within* the African diaspora as much as between Africans and other ancestries (42, 43). Why would Aboriginal populations have lower rates of AD than white Australians in rural environments, but have higher rates if they move to an urbanized environment (44)? How are heavily pigmented populations in India relatively immune to melanin’s theorized AD-inducing effects (43)? The authors state “African descendent individuals, as well as Asians and Pacific Islanders, are more likely to develop AD than Caucasian individuals” (6); but how does African descent cause AD in the African descendants living in urban America but not rural Africa? Why would being Caucasian be protective in Bulgaria but deleterious in Sweden (43)? If ancestry were a key factor, why would one’s birth home be more predictive of AD risk than one’s ancestral home (45)?

Even if one believed these questions could still be answered by innate biologic differences, how many Black Americans would you need to study to make statements about all Black Americans? Fifteen Black Manhattanites are unlikely to adequately represent all five New York boroughs, let alone comment on the disease for rural Black people in Alabama. Similarly, 15 white Manhattanites should not be framed as representative of all white Americans. Presenting these racialized claims in the context of a review of targeted therapies and personalized medicine suggests that the authors envision future practice parameters segregated into separate but equal treatment algorithms.

### 2.5 Genetics fail to explain racial disparities

Genetics do not explain racial disparities in AD (46, 47). The increased prevalence of AD in African American communities cannot be explained by: the allelic frequencies of *FLG* loss of function variants, copy number variations in *FLG*, the AD-polygenic score (PGS) derived from Europeans, the PGS for African ancestry, nor the PGS for pigmentation (46, 47). Taken together, the modern understanding of environmental exposures that contribute to AD require a baseline assessment of public health metrics prior to insinuating innate group differences, and especially before racializing such claims in atopy.

### 2.6 Using prior normalization to justify continuation

This manuscript was intended as a direct reply to Facheris et al. However, some may point to ongoing research into racial disparities writ large as defense of the authors’ claims. For example, the Journal of Clinical Medicine organized a special issue on “Ethnic differences in Dermatitis and Atopic Eczema and its Management” in March of 2023. Only three of the eight articles included in this special issue address differences between groups (12). One of these three citations focuses on the differences in presentation of AD in different skin colors and stresses the need to assure diverse patient cohorts in clinical trials (48). The second focuses on the environmental contributors to hand eczema that may differ by cultural practices (such as occupation or food preparation) (49). In stark contrast, the final example (12) also claims that Black American skin is devoid of Th17 cells and that European AD is distinct from Asian AD using the same flawed citations outlined above (6, 8, 18, 19). The paper (12) goes on to outline that Black patients should be given higher doses of cyclosporine by citing only an online news blog.

A recent report in *JACI in Practice* (50) echoed the claim that African American patients may require higher cyclosporine dosing. The 2004 review cited by JACI in Practice (51) enumerated three reports of higher cyclosporine metabolism among African Americans (52–54), two reporting no difference (55, 56), but overlooked a report of the opposite association between race and cyclosporine metabolism (57). Each of these studies enrolled patients being treated for solid organ transplants rather than AD. Each not only failed to assess a single social determinant of health, but also failed to adjust for factors known at the time to influence cyclosporine absorption such as diet, liver function, age, or concurrent medications (58). Once more, while the genetic variants referenced by modern studies as pharmacogenomic mechanisms for differing cyclosporine metabolism are not equally distributed across racial categories, race is not a functional proxy for genotyping (59). While some have attempted to argue that the correlation between race and social determinants of health indicate race is still a useful variable for statistical analysis (60), such practice represents a reliance on a flawed proxy of convenience in lieu of the effort needed to collect meaningful data. If the variable used could represent one of dozens different mechanisms (spanning sociology, psychology, hypothesized biochemistry, and more) then claims of using such information to design a targeted intervention (60) ring hollow.

Therefore, the correct phrasing would be to note that subjects with specific genotypes may require modulation of their cyclosporine dose in a race-neutral manner, as has been done successfully with other disorders (61). Doing so would accurately ascribe the need for dose modulation to the genotype, rather than racial category.

Others may defend these racialized practices by pointing to the National Institutes of Health’s (NIH) Request For Application (RFA). The assertions rest on the notion that NIH solicits research projects on racial disparities without explicitly prohibiting innate claims of biologic disadvantage in minoritized groups. To some, this is seen as tacit consent of the racialized differences in Th2 cytokine levels being valid. However, being open to further research into the mechanisms of racial disparities is not a defense of making sweeping claims about the biochemistry of the entire population of a continent using only data from one part of one nation. Claiming that a sixth generation Japanese American and someone who recently immigrated from rural China will both have similar Th22 expression based solely on their shared classification of Asian is definitionally incompatible with claims of seeing AD as a multifactorial disorder. If, however, Asian background is to be only one of many factors predicting drug response, these additional factors should be at least mentioned if not enumerated. Thankfully, a more recent review from the same group as Facheris et al. uses the more appropriate descriptor of “Japanese/Korean” instead of “Asian” (62), yet doing so continues to use the patients’ Japanese heritage as if it were a predictive variable while failing to evaluate the exposome-worth of variables that underlie the surrogate variable of ancestry.

Furthermore, openness to continued investigation does not answer the pointed questions of: what is the N value sufficient to study to justify claims of unique biology of pigmented skin?; how diverse of a cohort can be considered to be representative of Asia?; how comparable are studies that are performed years apart and using different equipment?; if a disease has identical symptoms, comorbidities, and treatment responses in populations all over the world, is it sound to predict that molecular causation would differ by skin tone?; and which environmental exposures are expected to be addressed for when evaluating AD across racial lines and national borders?

A related question would be to ask why innate biologic susceptibility to AD only manifested on a population scale after industrialization? Some have proposed that genetic variants that were beneficial in a pre-industrial era may have been rendered deleterious by exposures that were not common during human evolution (63). Yet, such framing centers disease causation on “the predisposed” in ways that mirror troubling post-WWII era of so-called “reform eugenics” (64). Even when the hypothesis of ancient DNA driving modern disease is put forward in good faith, proving such claims would require identification of the offending agent followed by mechanistic studies to verify the proposed gene–environment interaction. Furthermore, even if a toxin were shown to influence Th22 cell numbers via an allele more common among those of Han Chinese ancestry, avoidance of the toxin would remain paramount, and any imagined therapy would be targeted by genotype rather than ancestry.

### 2.7 Should ethical standards vary by impact factor?

One final justification of the types of claims that may be put forth by Facheris and Czarnowski is that journals which are subsidiary to the flagship publications of the publishing company should be more tolerant of claims based upon lower quality of evidence. Indeed, correlation between a journal’s prominence and its expected level of scientific veracity is a natural part of the scientific literature. However, as it applies to equity, tolerating poorly supported claims so long as they are limited to select journals suggests that the promises made by the publishers in the pages of the prestige journals were either applicable only to the lower tier journals or all together disingenuous. Furthermore, journal families are often distinguished by company logos, shared branding, and similar homepage websites. In an era of increasing concern for the potential harms of predatory journals (65, 66), branding is used by respected publishers to signal legitimacy to readers.

However, this branding is also used by bigoted online communities to endorse publications suggesting biologic determinism explains racial disparities. Although race science and eugenics are more commonly invoked for education attainment, social status, or mental health, research by several groups has demonstrated that racist online forums are the largest audience for publications purporting innate biologic differences explain racial disparities for common diseases (67–70). Such work also contributes to differential medical treatment through reinforcing the idea that biology differs between racial groups (71). Thus, all researchers should be mindful that racialized claims in their work may be dangerously misrepresented even when related to otherwise non-controversial topics like AD. Overall, a hereditarian view of AD proposes to improve care in ways that are theoretical and unlikely while provably providing aid and comfort to those wishing to advance marginalizing narratives.

### 2.8 Enumerating the impact of racialized claims

**Figure 1:**
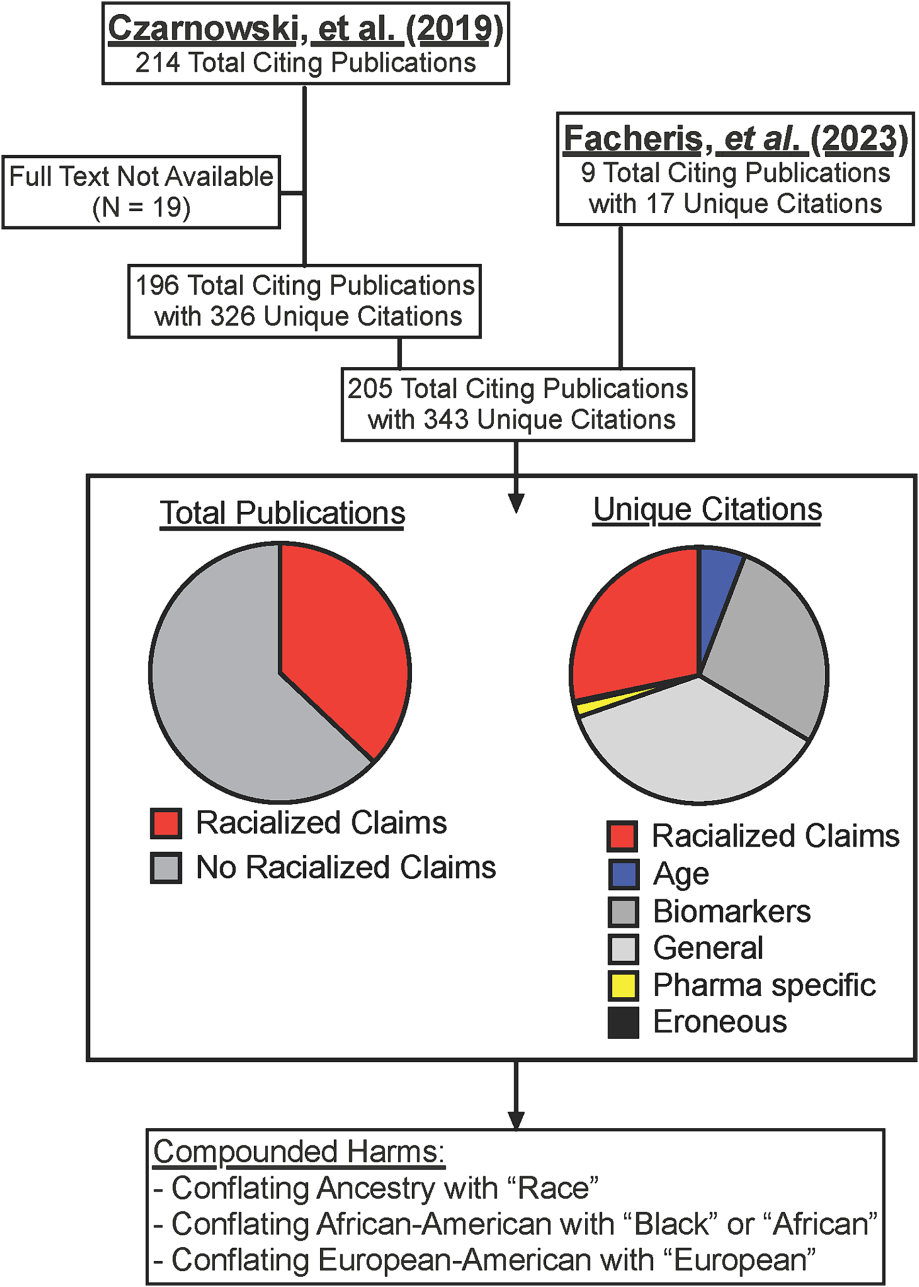
Dissemination of racialized claims in AD from two reviews. SCOPUS listed citations for Facheris (6) and Czarnowski (9) were collected and assessed for specific citations (some publications cited the articles more than once). Where full text was available, citations were assessed for racialized claims, or if the citation was referencing only age-related endotypes (Age), specific allergy cytokines of cells (Biomarkers), general comments on the existence of endotypes or AD symptoms (General), or comments on different prescription options in development (Pharma specific). Full citation list provided in Supplementary Table 1.

Per SCOPUS, Facheris (6) and Czarnowski (9) have been cited by a total of 224 publications (only 205 of which have full text availability) for a total of at least 343 unique citations within these publications (Supplementary Table 1). A plurality of the citations (36.2%) were general comments about AD pathogenesis or symptoms or non-specific references to the existence of presented endotypes (Figure 1). 27.7% of citations similarly referenced the specific biomarkers that may define the proposed endotypes such as Th17 versus Th22 cells. However, only 6 total citations (1.7%) from 6 publications (2.9%) were focused on the pharmaceutical discussion that was the stated intent of the Facheris (6) and Czarnowski (9) reviews. Instead, 28.2% of the total citations from 37% of the publications echoed the racialized claims made by the authors (Figure 1). The means that three studies which enrolled only 88 total people from the referenced groups became the basis for 97 references to the racialized AD endotypes for African Americans and “Asians” made by the Facheris (6) and Czarnowski (9) reviews. This calculation only includes first-level citations, and thus the 97 racialized claims citing Facheris (6) and Czarnowski (9) could themselves be used as citations in other papers. These harms were compounded when 11 of the publications used the terms “race” or “racial” rather than ancestry (72–82), 5 inappropriately extrapolated from “African American” to “Black” or “African” (83–87), and 3 made the same extrapolation from “European American” to “European” (88–90) (Supplementary Table 1).

## 3 Discussion

While the desire to avoid treating AD as “one size fits all” is noble, from a medical and biological perspective race is too imprecise to ever be included in “precision medicine” and too societally defined ever be appropriate for “personalized medicine.” It is likely that the authors, the reviewers, and the editors of the papers dissected herein never intended for their work to advance race science. However, extrapolating between exceedingly small cohorts and entire ancestry groups with an obliviousness to population-level environmental differences has direct ramifications for discussions of more controversial concepts. Overall, the various publishing groups will never be able to live up to the promise to avoid publishing race science until they recognize such work more often comes in the form of unintentional parroting of entrenched paradigms than overt statements of racial hierarchies. The scientific community must better adhere to reporting guidelines (21), avoid extrapolating small studies into population scales, assuring analyses are appropriately adjusted for social determinants, transparently reporting their study’s limitations, and prioritize the evidenced-based research into AD risk factors.

## Data availability statement

The original contributions presented in the study are included in the article/Supplementary material, further inquiries can be directed to the corresponding author.

## Author contributions

IM: Conceptualization, Data curation, Funding acquisition, Investigation, Methodology, Resources, Validation, Writing – original draft, Writing – review & editing.

## Footnotes

^1^ nationaleczema.org/visual-guide
